# Leveraging Social Media Activity and Machine Learning for HIV and Substance Abuse Risk Assessment: Development and Validation Study

**DOI:** 10.2196/22042

**Published:** 2021-04-26

**Authors:** Anaelia Ovalle, Orpaz Goldstein, Mohammad Kachuee, Elizabeth S C Wu, Chenglin Hong, Ian W Holloway, Majid Sarrafzadeh

**Affiliations:** 1 Department of Computer Science University of California Los Angeles Los Angeles, CA United States; 2 Department of Social Welfare University of California Los Angeles Los Angeles, CA United States

**Keywords:** online social networks, machine learning, behavioral intervention, data mining, msm, public health

## Abstract

**Background:**

Social media networks provide an abundance of diverse information that can be leveraged for data-driven applications across various social and physical sciences. One opportunity to utilize such data exists in the public health domain, where data collection is often constrained by organizational funding and limited user adoption. Furthermore, the efficacy of health interventions is often based on self-reported data, which are not always reliable. Health-promotion strategies for communities facing multiple vulnerabilities, such as men who have sex with men, can benefit from an automated system that not only determines health behavior risk but also suggests appropriate intervention targets.

**Objective:**

This study aims to determine the value of leveraging social media messages to identify health risk behavior for men who have sex with men.

**Methods:**

The Gay Social Networking Analysis Program was created as a preliminary framework for intelligent web-based health-promotion intervention. The program consisted of a data collection system that automatically gathered social media data, health questionnaires, and clinical results for sexually transmitted diseases and drug tests across 51 participants over 3 months. Machine learning techniques were utilized to assess the relationship between social media messages and participants' offline sexual health and substance use biological outcomes. The F1 score, a weighted average of precision and recall, was used to evaluate each algorithm. Natural language processing techniques were employed to create health behavior risk scores from participant messages.

**Results:**

Offline HIV, amphetamine, and methamphetamine use were correctly identified using only social media data, with machine learning models obtaining F1 scores of 82.6%, 85.9%, and 85.3%, respectively. Additionally, constructed risk scores were found to be reasonably comparable to risk scores adapted from the Center for Disease Control.

**Conclusions:**

To our knowledge, our study is the first empirical evaluation of a social media–based public health intervention framework for men who have sex with men. We found that social media data were correlated with offline sexual health and substance use, verified through biological testing. The proof of concept and initial results validate that public health interventions can indeed use social media–based systems to successfully determine offline health risk behaviors. The findings demonstrate the promise of deploying a social media–based just-in-time adaptive intervention to target substance use and HIV risk behavior.

## Introduction

Men who have sex with men are disproportionately affected by HIV and other sexually transmitted infections. In the United States, men who have sex with men accounted for two-thirds of incident HIV infections and more than half of new syphilis diagnoses in 2018 [1-3]. Substance use has been a persistent driver of the ongoing HIV epidemic in men who have sex with men. Research suggests substance use is highly associated with high-risk sexual behaviors such as condomless anal sex, multiple sex partners, and sex trading for drugs [4-6].

Web-based communication tools such as social networking sites (eg, “hookup apps,” dating websites) have been used among men who have sex with men to seek sexual partners and share information and resources about substance use [7-10]. In the early 2010s, 85% of men who have sex with men used the internet to find sexual partners [9], and this figure grew to 96% in 2019 [11]. However, the rising popularity of these technologies has also raised concerns about their role in facilitating sexual risk behaviors. Studies [12] have shown that men who have sex with men are more likely to engage in condomless anal sex with sex partners met online compared to partners met offline and have demonstrated that men who have sex with men who seek partners online have greater numbers of sexual partners compared to those who do not seek partners online. Furthermore, men who have sex with men who identify sexual partners online have a greater likelihood of substance use [13], although the evidence is equivocal [8,14,15]. Further studies are needed to provide empirical evidence of the association between online social networking technologies and offline sexual and substance use behaviors.

With more than 40% of health care consumers utilizing social media for their health-related decision making, social networks have indeed caught the attention of the public health domain [16]. Population-based analyses and in-person interventions are costly, both in time and resources [17]. Health spending is projected to grow at an average rate of 5.5% per year, totaling $6.0 trillion by 2027, nearly one-fifth of the United States gross domestic product [18]. Given these costs, the opportunity for high user engagement, and accessibility of social media data, social networks provide a new opportunity to public health. In recent years, social media have been employed in behavioral and public health research and has demonstrated its effectiveness in prevention, education, and treatment [19,20]. For instance, analyzing user activity on social media platforms has been an effective way to estimate the risk and time of HIV infection [21]. A separate study [22] found that the strength of associations in a social network, its network shape, and size are predictors of HIV and sexually transmitted infection risk.

Public health studies have also begun using social media platforms to understand and intervene in sexual health and substance use risk behaviors among men who have sex with men; however, these methodologies remain nascent in that they still rely on self-report and costly data collection as a means of developing and testing interventions [23-25]. Research demonstrates the need to utilize big data in social media and machine learning to understand communication and patterns about substance use and observe and predict real-time risk behaviors [26-28]. These strategies can inform just-in-time adaptive interventions [29] that are responsive to the individual technology use patterns of research participants; however, before deploying any social media–based intervention, the feasibility and efficacy behind employing such a modality for public health initiatives in risk reduction must be determined.

In practice, adaptive intervention systems are driven by an ability to determine health risks. Placed in the context of social media data, it is encouraging to learn that assessing health risk using textual sources has shown promising results in disease-specific risk evaluation and in identifying individuals at higher risk of depression and self-harm [30-32]. While text collection from electronic health records is extremely effective in determining a diagnosis, it is not a readily available resource for continuous risk assessment. Meanwhile, social media text data have the advantage of being abundantly available and cost-effective. While these data are not as domain constrained as clinical notes, they remain promising channels to explore for risk assessment.

Additionally, system interventions should be able to accurately evaluate when an individual is about to engage in a targeted health risk behavior with high probability followed by successfully reducing such behaviors. Maher et al [33] reviewed the effectiveness of past social network interventions, concluding with a call for stronger evidence in interventions that incorporate online social networks. Our paper responds to this call by evaluating the efficacy of social media data in determining health risk behavior. We are guided by the following questions: (1) Can we further substantiate the association between online social networking technologies and offline sexual and substance use behaviors? (2) Can we extract health risk scores from social media data that align with public health expert evaluation?

In this paper, the practicality of social media as an intervention modality is evaluated through social media data to identify health risk behavior in a sample of men who have sex with men from Los Angeles, California. The contributions of this paper are the following: (1) an end-to-end platform that continuously collects data from common social media platforms and specialized social networks tailored to the men who have sex with men community, and in tandem, biological data and personal health questionnaires were collected at baseline, 1-month, and 3-months from intake; (2) health behavior risk scores that are comparable to adapted risk scores created by the Centers for Disease Control and Prevention (CDC) using natural language processing techniques; and (3) the application of machine learning techniques to determine the extent to which social media messages can be used to directly predict verified biological outcomes of substance use and sexual risk, reflected as sexually transmitted disease diagnoses.

## Methods

### Study Protocol

**Figure 1:**
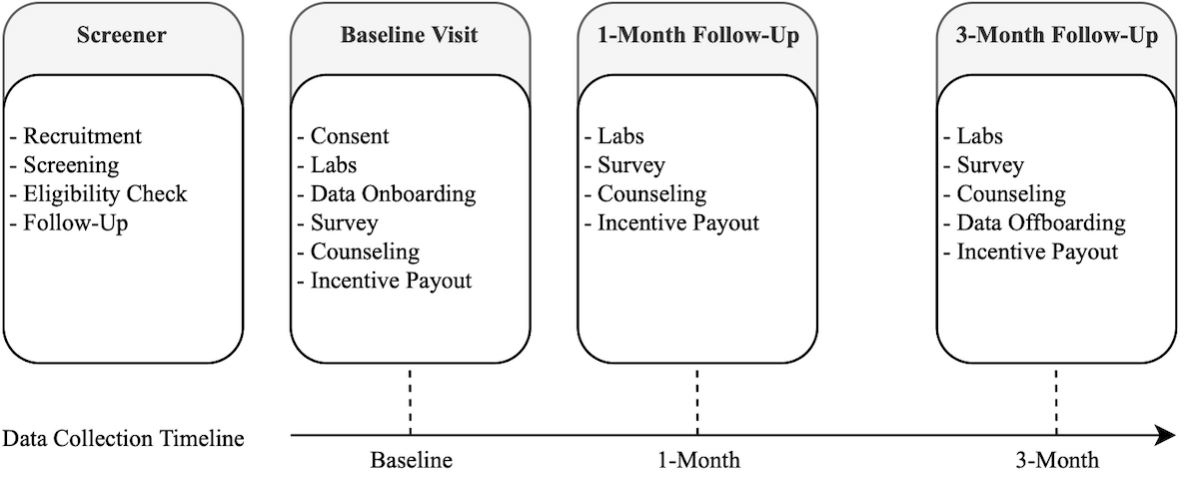
Different phases of the study protocol and data collection.

The study protocol consisted of 4 milestones: (1) screener, (2) baseline visit, (3) 1-month follow-up, and (4) 3-month follow-up (Figure 1). In the screener, flyers, online advertisements, and referrals were used to recruit potential participants. Further screening took place over the phone or through an web-based survey. Criteria such as age, sexual orientation, substance use, online dating, and social media activity were used to determine each participant's eligibility for the study.

Qualified participants were invited to an initial clinical visit to review the study in detail, ask for informed consent, and answer any of their remaining questions. Afterward, a series of lab tests were conducted to determine their substance use and the presence of sexually transmitted diseases. Site testing was conducted for *Chlamydia trachomatis* and *Neisseria gonorrhoeae* with pharyngeal, urethral, and anal swabs. Further tests included a rapid plasma reagin blood test for syphilis, a rapid oral test for HIV, and a urine drug screen. Additionally, a survey was completed by the participants which asked a series of questions regarding demographic characteristics, sexual risk behavior, illicit substance use, and online behavior. Finally, participants provided their log-in credentials for a set of social media sites on which they had been. The user credentials were registered in a custom data collection platform for each website and the participants authorized the data collection system to pull their daily online activity. We collected participant social media data for up to 3 months after onboarding. We found this to be a reasonable duration considering the need to follow participants long enough to observe any changes in social media use and behaviors over time that can be measured by follow-up surveys and their biomarkers.

The system began collecting participants' daily social media activity immediately after the baseline visit. One month into the study, they were scheduled to revisit the clinic and redo lab tests and surveys. A final follow-up was set for 3 months after the baseline visit to recollect lab and survey data in addition to conducting required off-boarding procedures, including the discontinuation of participant data collection.

This study protocol was approved by the University of California, Los Angeles institutional review board (IRB 17-000408). Informed consent was obtained from all individual participants included in the study. Each participant was provided with up to US $150 in cash incentives based on their participation. Certain medical conditions identified within this study were also reported to appropriate agencies as required by federal and state laws. Proper consultation and referrals were provided to each participant before and after reporting each screening test.

### System Architecture

Data from Facebook and Twitter was automatically collected through official application programming interfaces (API). The APIs were used to query the content (eg, messages, posts) generated by each participant; however, it is important to note that Facebook changed its API policy during the study. Standard permission requests could no longer be used to access user data. To mitigate this issue and re-enable the permission to read user content, the participants enrolled as testers of a custom Facebook app developed during the study.

**Figure 2:**
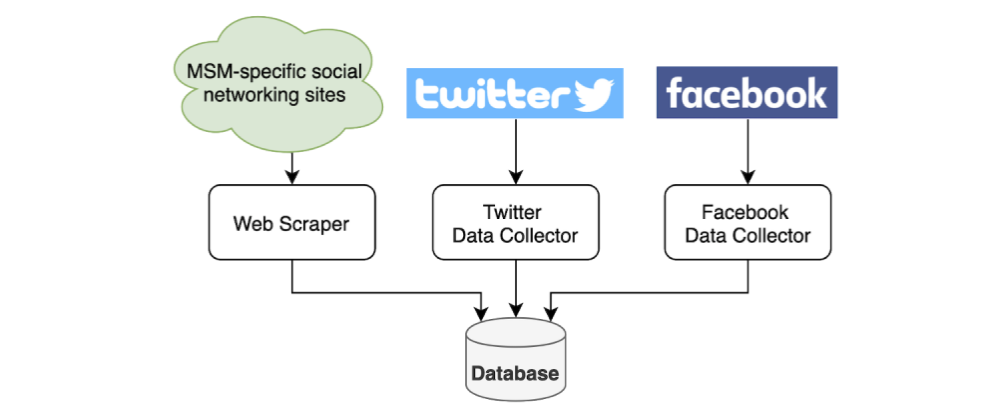
A visualization of the database schema used in this study. MSM: men who have sex with men.

In addition to these major social media platforms, we collected similar data fields for men who have sex with men–specific social networks. The software implementation slightly differed from that of Facebook and Twitter in that a unique web scraper was built to collect data. Due to the study's privacy protocol, the name of each site was omitted. The websites, labeled Website A and Website B, are one of the most established websites in the men who have sex with men community, with over 10 million users overall. Qualitative interviews and input from a community advisory board further informed the decision to include these websites. Each scraper used a combination of browsing automation tools and web parsers to mimic a user going online and accessing their profile. URL requests were made using Selenium, a browsing tool that can conduct automatic authentication, search, and navigate each site of interest [34]. Participants explicitly provided consent and their usernames and passwords per institutional review board protocol approvals. These credentials were used by Selenium to automate logging into a website and navigating to pages where the system was able to collect profiles and messages between users. The data were extracted from each website of interest using the BeautifulSoup Python Package and later saved to the database [35]. Figure 2 provides a visualization of the study's overall database schema. MySQL, a free and open-source database server, was used to store and query user messages and metadata including usernames and respective access tokens from each website. To maintain high data-quality, a hashing of the data and timestamp was created within the database to prevent duplication.

We validated that the system collected participant messages consistently and reliably by maintaining a group of test users. Before and throughout the study, various mixed-media data were sent across these platforms to make sure all types of generated data were correctly collected and accurately represented. The data consisted of text, videos, and emojis.

Due to the sensitive nature of collecting online social media activity, providing security and privacy was of paramount importance. Therefore, the data collection system was designed to offer protection on multiple levels: at the system-level and the data-level. At the system-level, the user data were stored on a dedicated private server behind a firewall, protecting against outside cyber-attacks and malicious software. At the data-level, the user data went through a data sanitation process. Participants' identifiers in the data collection system were anonymized and could only be matched with a reference datasheet stored externally on a cloud storage service compliant with the Health Insurance Portability and Accountability Act (HIPAA). The cloud storage service was also used to store all other data, such as participant questionnaires and drug screening results.

### Defining Health Behavior Risk

Given the ambiguous nature of identifying health risk behavior in social media messages, a map between commonly used colloquial terminology and risk behavior topics was curated. To gain a more nuanced understanding of online strategies and behaviors around seeking drugs and sexual partners, 24 men who have sex with men community members in Los Angeles who self-disclosed risky sex, illicit drug use, and usage of dating apps were qualitatively interviewed. The response to the following question became the foundation for the risk behavior dictionary: “What are some of the terms that you use on websites, chatrooms, message boards, and apps to find drugs, drug use partners, and/or sex partners?” The words were organized into topics (Table 1). All terms were vetted by a community advisory board throughout the study. The resulting risk behavior dictionary was leveraged to facilitate the modeling of the relationship between social media messages and health behavior risk.

Sexual health and illicit drug use risk was also evaluated for each participant using the CDC Risk Assessment Tool [36]. Each assessment was adjusted based on participants' weekly text message diary responses on illicit substance use. Scores for each participant were available at baseline intake, 1-month, and 3-month checkpoints.

**Table 1:** Sexual and substance use topics associated with colloquial terms found in text conversations based on qualitative interviews with men who have sex with men community members.

Topic	Words
Alcohol	drunk, drinking, booze, party, liquor
Marijuana	toke, pipe, weed, pot, mary jane
Cocaine	crack, blow, snow, yayo, powder
Methamphetamine	meth, speed, ice, crank, crystal
Amyl nitrate	poppers, rush, pops, amyl
Heroin	dope, smack, junk, tar
Ecstasy	X, Ex, molly, rolling, mdma
GHB	G, roll, water
Ketamine	K
Type of substance other/general	pills, favors, DMT, party, party favors
Snorting	snort, sniff, rack, rail, lines
Inhaling	smoke, blow clouds, hit, puff
Injection	straight to the point, straight to the poinT, slam, slamming, shoot, shooting
Anal insertion	booty bumping, butt rocket, plug, plugging
Substance use behavior other/general	gen, generous, friends with benefits
Buying drugs	Do you have a connect, I can contribute, can you do me a big/little/huge favor
Masturbation	JO, jack, stroke, HJ, jack off
Oral sex	blow, head, gloryhole, suck, BJ
Anal intercourse	top, bottom, fuck, power top, power bottom
Group sex	3some, 3way, gang bang, orgy, bukkake
Sex work	$, roses, generous, pro, GEN
Anonymous	anon, discreet, discrete, anonymous, random play
Sex with condoms	condoms, rubber, safe sex, play safe, safe
Condomless sex	Bareback, bare, raw, seed, seeding
Substance use and sex	Party and play, smoke and stroke, pnp, party, partying
Sexual behavior general/other	69

### Data Set Processing and Auditing

Data were cleaned to maximize consistency and accuracy. The messages were first screened using Python regular expressions to pattern match texts identified as spam or in-app advertisements. Pattern personal identifiable information such as addresses and phone numbers were tokenized. For instance, if a phone number was provided in a message, it was replaced with “phonenumbertoken.” Data were deduplicated and the privacy of those that communicated with our participants was protected. This was done by only considering the participants' sent messages, resulting in data for 48 individuals. Some messages also consisted of notifications such as when a participant's profile was seen, clicked on, or had a request to unlock their photos—all of which were tokenized with the suffix “token.” One data source in the study was excluded because it failed to produce data due to changes in the site's data collection policy. Furthermore, we focus our analysis on biomarkers that were present in at least 10% of participants.

After data cleaning, the messages went through an automated pattern matching pipeline using regular expressions to identify terms in the risk behavior dictionary defined in Table 1. The mapping was utilized to flag words and colloquial terms associated with health risk behaviors in each message. As an example, if the word in a given message was “rail,“ it was matched to the topic of snorting.

### Biomarker Prediction With Social Media Messages

The relationship between participants' social media behavior and illicit substance use and sexual risk behaviors was examined; data collected across social media accounts were leveraged to predict participant’s respective offline substance use and sexual health biomarkers.

Standardized counts of each tokenized word and text-summary features, such as message length, were used as simple features to predict drug use and sexually transmitted diseases. Each outcome was treated as its own binary classification task. Logistic regression, linear support vector machine, naïve Bayes, and random forest models were employed to predict the outcomes at 1-month and 3-month follow-up. Given the relatively small data set and the challenge of class imbalance, stratified 5-fold cross-validation was used to assess the generalizability of the predictive models. The performance of each model was assessed using the precision, defined as *true positive* / (*true positive + false positive*); recall, defined as *true positive* / (*true positive + false negative*); and F1 score, defined as 2 × *precision* × *recall* / (*precision + recall*).

### Message-Based Risk Scores

Health risk behavior was assessed on a per-message level using available social media text correspondence. A risk score was given for each message based on how likely its words were associated with those in the risk behavior dictionary.

### Identifying Health Risk Behaviors Using Social Media Messages

Natural language processing techniques were employed to create a risk score using social media data. We employed a Skip-Gram Word2Vec model which allowed us to extract word representations to determine the association between words in social media messages and words in the risk behavior dictionary [37]. The Skip-Gram Word2vec model constructs a word representation, or word embedding, based on how well it predicts the words that surround it within a given radius. Given all the words in the message corpus, *w*_1_, *w*_2_, *w*_3_,..., *w*_T_, the model tries to predict the probability of observing the context word *w*_t+j_ given a target word *w*_t_.



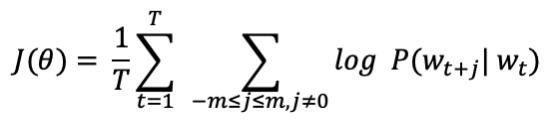



The resulting model constructs a vector space that identifies context-dependent similarity between words used by the participants in the selected social networks monitored.

### Health Behavior Risk Scoring

Once contextually similar instances of risky text messages are found, a risk score is constructed with cosine similarity, similar to the approach used by Kiros et al [38]. Given user *i*’s *t*th message, the risk score is defined as the average cosine similarity between the word in the message, 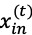, and the risky word *w* from each risk topic *d* set of risky words.



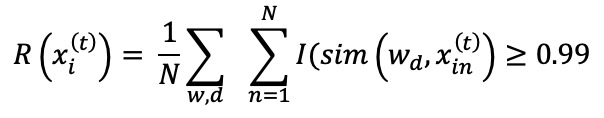



The distance between the words found in messages and those from the risk behavior dictionary was used to decide when a user was displaying a risky textual correspondence. Afterward, similarity between the risk scores generated by the model and those provided by the CDC risk assessment tool was assessed for each participant.

## Results

### Data

**Figure 3:**
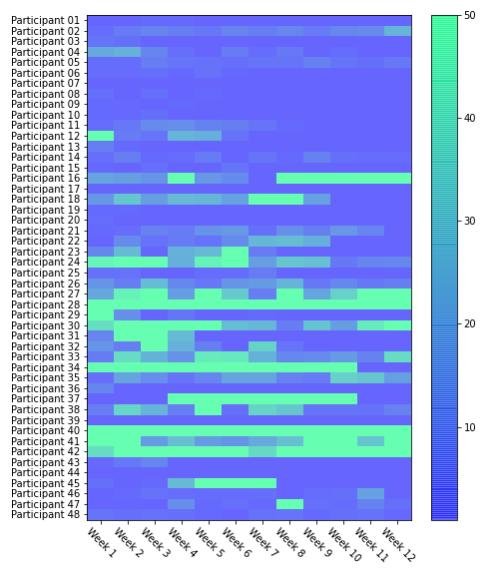
A heatmap of user activity across all sources of data during the study.

A total of 15,695 sent messages were collected in the 3-month timeline for 48 participants across 4 different platforms: Twitter, Facebook, Website A, and Website B after preprocessing. 6.5% (1026) of the messages were advertisements filtered out by our data processor. As shown in Table 2, the majority of messages, 75.7% (11,877), came from Website A, followed by Website B with 21.2% (3327), Facebook at 2.2% (352), and Twitter with 0.89% (139) of messages. Participant activity across all platforms is displayed as a heatmap in Figure 3, with participants 28, 40, and 42 showing the highest activity after initial onboarding.

**Figure 4:**
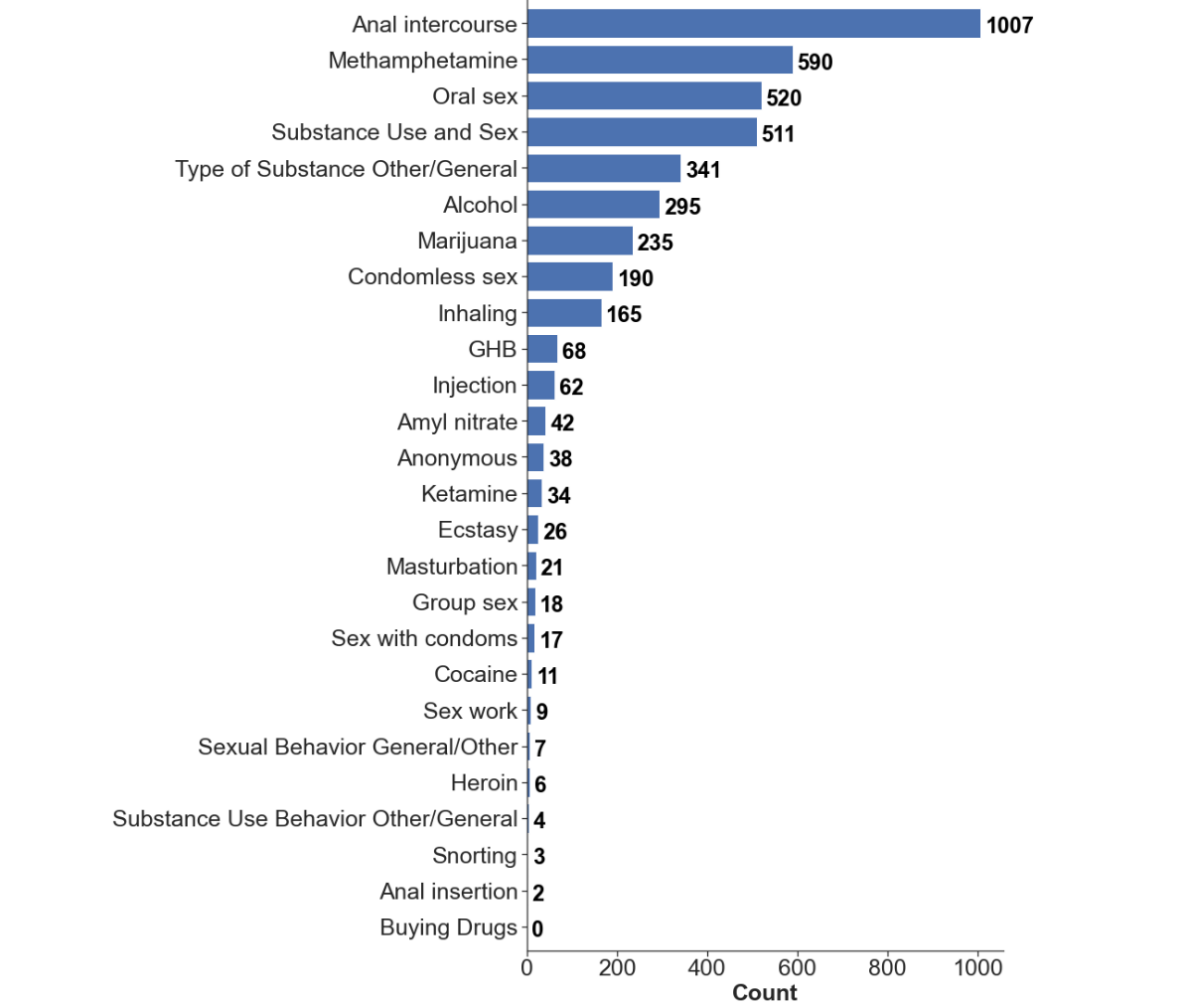
Topics found in messages using the risk behavior word dictionary provided by public health experts.

The distribution of each topic is visualized in Figure 4. The topics with the most discussion across the social media sites were anal intercourse and methamphetamines use. In the messages themselves, tokens such as “clickedprofiletoken,” “unlockedphotostoken,” and “phonenumbertoken” were the highest occurring tokens across all messages. This makes sense, as activity in dating sites is heavily based on interacting with photos and other information exchange, potentially leading up to an offline connection.

In terms of clinical data, the distribution of each outcome can be seen in Table 3, where the majority of selected outcomes suffer from a 10% to 15% imbalance.

**Table 2:** Messages sent across social media platforms by participants.

Source	Messages sent, n (%^a^)	Cumulative messages sent, n (%^a^)
Twitter	139 (0.9)	139 (0.9)
Facebook	352 (2.2)	491 (3.1)
Website^b^ A	3327 (21.2)	3818 (24.3)
Website^b^ B	11,877 (75.7)	15,695 (100)

^a^Percentage of total messages.

^b^Specific to men who have sex with men.

**Table 3:** Distribution of biomarker outcomes in a 1-month and 3-month follow-up after participants’ initial onboarding.

Test and outcome				1-month follow-up (n=48), n (%)		3-month follow-up (n=48), n (%)
**Sexually transmitted disease**						
	**HIV^a^**					
		Positive	20 (41.7)		23 (47.9)	
		Negative	28 (58.3)		25 (52.1)	
**Substance use**						
	**Amphetamine**					
		Positive	24 (50.0)		21 (43.8)	
		Negative	24 (50.0)		27 (56.2)	
	**Methamphetamine**					
		Positive	25 (52.1)		22 (45.8)	
		Negative	23 (47.9)		26 (54.2)	
	**THC^b^**					
		Positive	20 (41.7)		18 (37.5)	
		Negative	28 (58.3)		30 (62.5)	

^a^HIV: human immunodeficiency virus.

^b^THC: tetrahydrocannabinol.

### Biomarker Prediction

Of the 4 biomarkers, only 3 reflect an F1 score greater than 80%—HIV, amphetamine, and methamphetamine (Table 4). Although the tetrahydrocannabinol outcome did not suffer from severe class imbalance, one reason that may explain the significantly poorer performance is that the topic was heavily impacted by polysemy. As an example, participants alluded to marijuana usage with words and phrases that had multiple meanings. The phrase “blowing clouds” could refer to smoking marijuana or smoking methamphetamines.

For these 3 offline biomarker outcomes, the random forest model resulted in the highest F1 scores. Amphetamine usage was the best predicted outcome for all 4 models.

**Table 4:** F1 scores for each respective model and outcome in a 1-month and 3-month follow-up period after participants’ initial onboarding.

Outcome and model		F1 Score, %	
		1-month follow-up	3-month follow-up
**HIV^a^**			
	Support vector machine	70.5	81.6
	Random forest	73.4	82.6
	Naïve Bayes	69.6	82.1
	Logistic regression	68.5	81.4
**Amphetamines**			
	Support vector machine	88.2	85.9
	Random forest	88.3	85.9
	Naïve Bayes	88.0	85.5
	Logistic regression	88.1	85.8
**Methamphetamines**			
	Support vector machine	88.3	85.1
	Random forest	88.3	85.3
	Naïve Bayes	88.1	84.8
	Logistic regression	88.2	85.0
**THC^b^**			
	Support vector machine	11.1	7.4
	Random forest	5.0	0.5
	Naïve Bayes	24.0	4.7
	Logistic regression	10.9	9.1

^a^HIV: human immunodeficiency virus.

^b^THC: tetrahydrocannabinol.

### Associating Words With Health Behavior Risk

**Figure 5:**
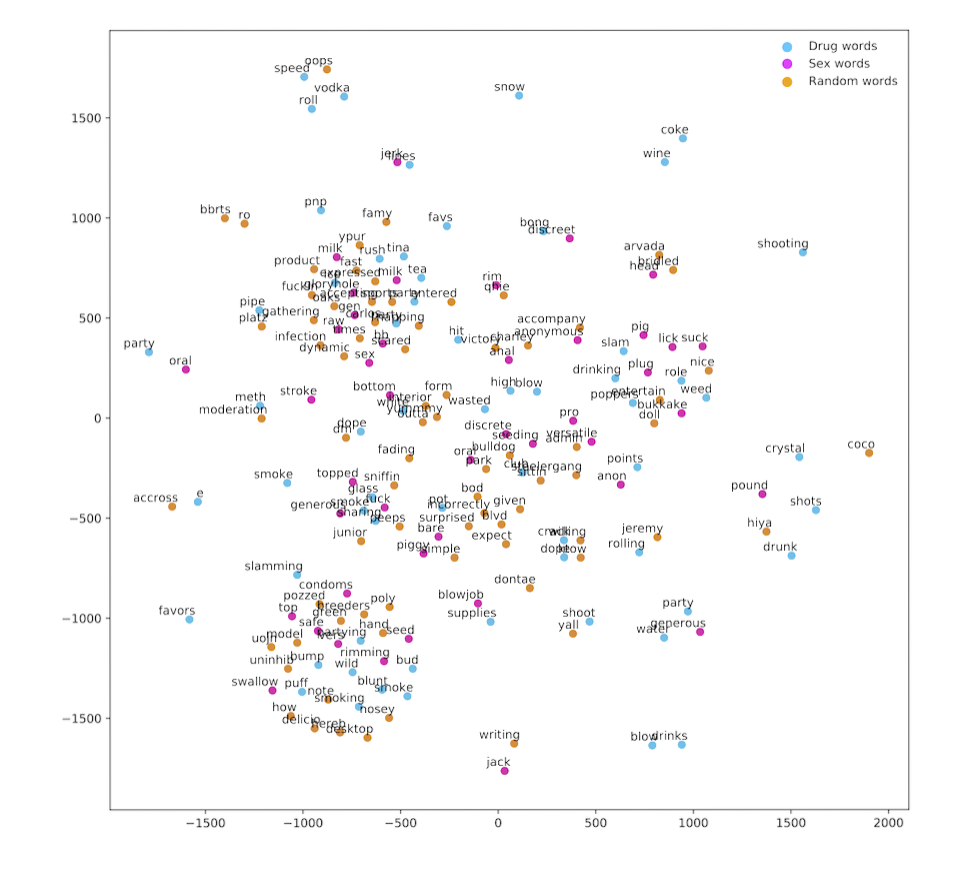
A t-distributed stochastic neighbor embedding visualization of the resulting vector space after running Word2Vec: drug-related words, sex-related words, and randomly chosen words.

A dimensionality reduction technique, *t*-distributed stochastic neighbor embedding, was used to observe the relationship between word embeddings [39]. Figure 5 shows the resulting vector space as words are projected onto a 2D plane. Intuitively, the resulting proximity between words can be interpreted as their similarity. As a result, natural groupings between drug and sex-related words form. For instance, the word “Smurff,” a term used to describe oral sex, is grouped with the term “head,” a known colloquial term for oral sex.

**Figure 6:**
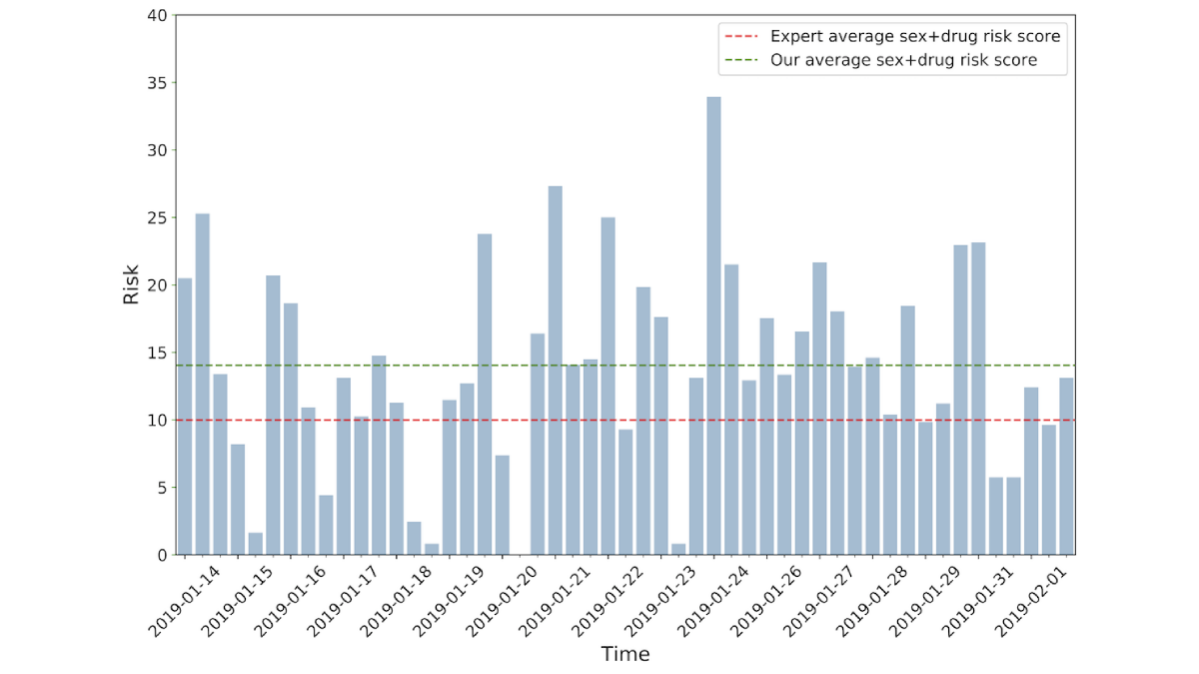
Normalized expert risk evaluation for drug and sex at 1 month and 3 months.

Health behavior risk scores were calculated using the Word2Vec word vector space. Risk scores were created each day for a given user in addition to their average risk score along with risk scores provided by public health experts. While the constructed daily scores vary across time, there is a visible similarity between the generated risk score and expert scores, on average (Figure 6).

## Discussion

### Principal Results

To our knowledge, our study is the first empirical evaluation of a social media–based public health intervention framework for men who have sex with men. Our qualitative work highlighted the ways in which men who have sex with men use coded language online to refer to specific substance use behavior and HIV risk. We investigated the association between social media data and offline health risk behavior by operationalizing social media data across several networking sites. We built a system that automated social media data collection, which allowed us to predict offline substance use and sexual health biomarkers and construct daily health behavior risk scores.

In conducting an exploratory data analysis to check for data quality, we validated that the system was indeed able to collect participant messages consistently and reliably using the proposed system architecture. It was reasonable to observe that most messages came from men who have sex with men–specific social networks, as individuals may be more reserved around topics related to health risk behaviors over platforms with a larger audience such as Facebook and Twitter. Furthermore, we observed that after the topic of anal intercourse, the topic of methamphetamines was the most frequent in risk behavior conversation (Figure 5).

We tested the extent to which social media data could provide meaningful insight into health risk behaviors by predicting offline sexual health and substance use biomarkers. We found that across the models and clinical timelines, there was a consistently high F1 score when predicting HIV, amphetamine, and methamphetamine use. These results are validated by the fact that methamphetamines are one of the most commonly used drugs in among men who have sex with men [5]. Moreover, these findings align with existing literature pointing to the association between methamphetamine use and increased risk for HIV [4-6,10,13]. Most importantly, we validate the significance of social media data in its association with substance use and sexual health biomarker outcomes.

Daily health risk scores were created for participants using only social media data. The method was validated by the observation that the constructed risk scores, on average, were comparable to adapted risk scores created by the CDC risk assessment tool. The results were promising at a fundamental level, as we observed how words in social media messages can indeed cluster with known drug and sex-related words when mapped to a vector space (Figure 5). In constructing an average risk score for participants, we used their text messages and respective contexts to extract risk; we assumed that the public health risk dictionary was most accurate and encompassing of health risk behavior terminology. The health behavior risk scores were constructed on a daily level. Only social media data were used to construct the risk score, while public experts used biologically verified results and self-reported data to construct monthly risk scores by adapting a version of the CDC risk assessment tool. Yet, even with this significant difference in methodology, the average risk was still quite similar to the expertly assessed risk. This suggests that the community-based participatory approach used to create the data dictionary was crucial to the risk score creation process. Hence, the success of using such an approach heavily relies on involving end users in the creation of social media tools.

### Limitations

The technical setup and maintenance needed for a social media data collection platform is an important consideration for scalability. Extensive effort and resources were required to achieve HIPAA compliance for our system. Additionally, creating the data collector required custom handling of each data source. For instance, Facebook's policy change called for a completely different approach to data collecting in the middle of the study. To provide a scalable solution, the data collection platform should be flexible enough to adjust to a new collection regime due to circumstances outside of the study's control.

Several outcomes did not pass our exclusion criteria filter at the beginning of the study due to a significant imbalance in their respective distributions, leading to challenges for biomarker prediction. Additionally, employing methods that handle class imbalance may improve our current F1 scores. At a higher level, it is also important to note that the scope of this paper is somewhat limited since we only collected data from a few websites, which may not be used by the entire community of men who have sex with men.

### Implications

Self-report is a common form of feedback for health behavior interventions. While useful, it is often flawed because of biases such as recall bias and social desirability bias. Therefore, the health behavior data in this study were combined with biologically verified data to calculate risk. We found that social media may serve as a valid intervention modality as it provides both valuable and relevant feedback. Overall, we determined that combining self-report data with biologically verified outcomes and social media data mining gives a more nuanced and accurate picture of health behavior risk. These findings suggest that it is feasible to use social media data for future public health intervention.

### Conclusions

In this study, we created an end-to-end system that leverages social media data for health behavior risk identification, serving as a proof of concept for social media–based behavioral intervention. We demonstrated that it is possible to build an integrative system across multiple platforms that effectively collects meaningful social media data. We determined that social media messages are a valuable source of examining the relationship between health risk behaviors and biologically verified sexual diseases such as HIV and illicit usage of amphetamines and methamphetamines among men who have sex with men. Adapted CDC health risk scores were compared against social media–based behavioral risk scores and found to be, on average, similar to the expertly assessed scores. This validates the feasibility of employing a social media–based behavioral intervention. The contributions made in this paper are stepping stones toward building an automated, cost-effective, fully scalable social media intervention system that serves the public health domain.

## Abbreviations

API: application programming interface
CDC: Centers for Disease Control and Prevention
HIPAA: Health Insurance Portability and Accountability Act
HIV: human immunodeficiency virus

## References

[1] (2019). 2018 STD surveillance report. Centers for Disease Control and Prevention.

[2] HIV surveillance reports. Centers for Disease Control and Prevention.

[3] Purcell DW, Johnson CH, Lansky A, Prejean J, Stein R, Denning P, Gau Zaneta, Weinstock H, Su J, Crepaz N (2012). Estimating the population size of men who have sex with men in the United States to obtain HIV and syphilis rates. Open AIDS J.

[4] Buchacz K, McFarland Willi, Kellogg T, Loeb Lisa, Holmberg Scott D, Dilley James, Klausner Jeffrey D (2005). Amphetamine use is associated with increased HIV incidence among men who have sex with men in San Francisco. AIDS.

[5] Hoenigl M, Chaillon A, Moore D, Morris S, Smith D, Little S (2016). Clear links between starting methamphetamine and increasing sexual risk behavior. J AIDS.

[6] Berg RC, Weatherburn P, Marcus U, Schmidt AJ (2019). Links between transactional sex and HIV/STI-risk and substance use among a large sample of European men who have sex with men. BMC Infect Dis.

[7] Grov C, Breslow AS, Newcomb ME, Rosenberger JG, Bauermeister JA (2014). Gay and bisexual men's use of the internet: research from the 1990s through 2013. J Sex Res.

[8] Bien CH, Best JM, Muessig KE, Wei C, Han L, Tucker JD (2015). Gay apps for seeking sex partners in China: implications for MSM sexual health. AIDS Behav.

[9] Rosser BRS, Wilkerson JM, Smolenski DJ, Oakes JM, Konstan J, Horvath KJ, Kilian GR, Novak DS, Danilenko GP, Morgan R (2011). The future of Internet-based HIV prevention: a report on key findings from the Men's INTernet (MINTS-I, II) Sex Studies. AIDS Behav.

[10] Bourne A, Reid D, Hickson F, Torres-Rueda S, Weatherburn P (2015). Illicit drug use in sexual settings ('chemsex') and HIV/STI transmission risk behaviour among gay men in South London: findings from a qualitative study. Sex Transm Infect.

[11] Shrestha R, Lim SH, Altice FL, Copenhaver M, Wickersham JA, Saifi R, Ab Halim MA, Naning H, Kamarulzaman A (2020). Use of smartphone to seek sexual health information online among Malaysian men who have sex with men (MSM): implications for mHealth intervention to increase HIV testing and reduce HIV risks. J Community Health.

[12] Grosskopf NA, LeVasseur MT, Glaser DB (2014). Use of the Internet and mobile-based "apps" for sex-seeking among men who have sex with men in New York City. Am J Mens Health.

[13] Taylor MM, Aynalem G, Smith LV, Montoya J, Kerndt P (2007). Methamphetamine use and sexual risk behaviours among men who have sex with men diagnosed with early syphilis in Los Angeles County. Int J STD AIDS.

[14] Liau A, Millett G, Marks G (2006). Meta-analytic examination of online sex-seeking and sexual risk behavior among men who have sex with men. Sex Transm Dis.

[15] Zhang D, Bi P, Lv F, Zhang J, Hiller JE (2008). Differences between internet and community samples of MSM: implications for behavioral surveillance among MSM in China. AIDS Care.

[16] Warden C ReferralMD.

[17] Pagoto S, Waring ME, May CN, Ding EY, Kunz WH, Hayes R, Oleski JL (2016). Adapting behavioral interventions for social media delivery. J Med Internet Res.

[18] (2020). Health care costs 101: spending keeps growing. California Health Care Foundation.

[19] Lemley SM, Klausner JD, Young SD, Stafylis C, Mulatya C, Oden N, Xie H, Revoredo L, Shmueli-Blumberg D, Hichborn E, McKelle E, Moran L, Jacobs P, Marsch LA (2020). Comparing web-based platforms for promoting HIV self-testing and pre-exposure prophylaxis uptake in high-risk men who have sex with men: protocol for a longitudinal cohort study. JMIR Res Protoc.

[20] Rhodes SD, McCoy TP, Tanner AE, Stowers J, Bachmann LH, Nguyen AL, Ross MW (2016). Using social media to increase HIV testing among gay and bisexual men, other men who have sex with men, and transgender persons: outcomes from a randomized community trial. Clin Infect Dis.

[21] Christley R, Pinchbeck G, Bowers R, Clancy D, French N P, Bennett R, Turner J (2005). Infection in social networks: using network analysis to identify high-risk individuals. Am J Epidemiol.

[22] Drumright LN, Frost SDW (2010). Rapid social network assessment for predicting HIV and STI risk among men attending bars and clubs in San Diego, California. Sex Transm Infect.

[23] Holloway IW, Pulsipher CA, Gibbs J, Barman-Adhikari A, Rice E (2015). Network influences on the sexual risk behaviors of gay, bisexual and other men who have sex with men using geosocial networking applications. AIDS Behav.

[24] Holloway IW (2015). Substance use homophily among geosocial networking application using gay, bisexual, and other men who have sex with men. Arch Sex Behav.

[25] Wang H, Zhang L, Zhou Y, Wang K, Zhang X, Wu J, Wang G (2018). The use of geosocial networking smartphone applications and the risk of sexually transmitted infections among men who have sex with men: a systematic review and meta-analysis. BMC Public Health.

[26] Centola D (2011). An experimental study of homophily in the adoption of health behavior. Science.

[27] Eichstaedt JC, Smith RJ, Merchant RM, Ungar LH, Crutchley P, Preoţiuc-Pietro D, Asch DA, Schwartz HA (2018). Facebook language predicts depression in medical records. Proc Natl Acad Sci U S A.

[28] Kalyanam J, Katsuki T, R G Lanckriet Gert, Mackey Tim K (2017). Exploring trends of nonmedical use of prescription drugs and polydrug abuse in the Twittersphere using unsupervised machine learning. Addict Behav.

[29] Platteau T, Herrijgers C, de Wit J (2020). Digital chemsex support and care: the potential of just-in-time adaptive interventions. Int J Drug Policy.

[30] Feller D, Zucker J, Yin M, Gordon P, Elhadad N (2018). Using clinical notes and natural language processing for automated HIV risk assessment. J AIDS.

[31] Korhonen A, Séaghdha Diarmuid O, Silins I, Sun L, Högberg Johan, Stenius U (2012). Text mining for literature review and knowledge discovery in cancer risk assessment and research. PLoS One.

[32] Yates A, Cohan A, Goharian N (2017). Depression and self-harm risk assessment in online forums. Proceedings of the 2017 Conference on Empirical Methods in Natural Language Processing.

[33] Maher CA, Lewis LK, Ferrar K, Marshall S, De Bourdeaudhuij Ilse, Vandelanotte C (2014). Are health behavior change interventions that use online social networks effective? a systematic review. J Med Internet Res.

[34] Selenium. Github.

[35] Richardson L (2007). Beautiful Soup documentation. Crummy.

[36] HIV risk reduction tool. Centers for Disease Control and Prevention.

[37] Mikolov T, Sutskever I, Chen K, Corrado G, Dean J (2013). Distributed representations of words and phrases and their compositionality. Proceedings of Advances in Neural Information Processing System.

[38] Kiros R, Salakhutdinov R, Zemel R (2014). Unifying visual-semantic embeddings with multimodal neural language models.

[39] Maaten L (2008). Visualizing data using t-SNE. J Mach Learn Res.

